# Professional Society

**Published:** 1991-09

**Authors:** Jack Davies


### From our Correspondents

### PROFESSIONAL SOCIETY

Both the National Health Service and Universities seem to be in a mess, unsure whether they are providing services or fulfilling commercial roles. I am still not sure whether this piece really ought to be a book review or a comment in the Correspondent's Column. It concerns the essence of the thesis proposed by Harold Perkin, a well known social historian who now works in the United States after academic posts during the 1960s and 1980s at Manchester and Lancaster. So confused are we all, that it could be that steering through nearly 600 pages of text is worth while.[1]

Historically feudalism and a landowning aristocracy throughout Europe proved unable to cope with the rise of the expanding and commercial system of towns and cities. [2,3] In the great wide world there arose two mutually antagonistic systems: entrepreneurial capitalism and organised labour. Perkin further suggests that with the establishment of an effective educational network, there also appeared a "new" professional class which fitted into neither system. Sceptics will understand that this process has taken place since the arrival of the professional clerk. None the less, since 1880 this professional class has in his view taken over the organisation of both systems. No class is uniform, and Perkin's professionals have many subtle distinctions and rivalries.

So far so good. As a test of the hypothesis, just try complaining yourself about municipal garbage removal, changing an airline booking or getting through the customs. There is little chance of speaking directly to the boss dustman, the airline owner or the President of the country; you are faced by the professionals. Patients face the same panoply of dragons-at-the-gate. Have you ever walked into a chemist's shop wanting to buy oral penicillin whilst on holiday? It's the same story.

Understanding history is often said to help solving current problems. Personally, I suspect that it tells us merely how the problems have arisen. Even that, however, may be a solace.

Back to the present. What is not addressed in Perkin's book is the conflict of interests which become apparent when professionals attempt to be both representative of organised labour and at the same time to become minor capitalists. The new philosophy of the National Health Service, and of the universities, seems to be trying to impose just this. Is a bum but profitable MSc course, or a financially "efficient" but clinically dangerous service what we all want?

Possibly our east European professional colleagues don't quite see it the same way.[4] Nevertheless, they will probably influence our thinking now that European boundaries are fast disappearing. Is this the post-Thatcherite revolution in sight?

Jack Davies

### REFERENCES

1. PERKIN H. *The rise of professional society: England since 1880.* London: Routledge. 1990.
2. BRAUDEL F. *L'identité de la France: les hommes et les choses.* Vol. III. Paris: Arthaud-Flammarion. 1986.
3. THOMPSON F.M.L. *English landed society in the nineteenth century.* London: Routledge & Kegan Paul. 1963.
4. DEMPSEY J, COLITT L, DENTON N. et al. The other Europeans on the move. *The Financial Times.* 1991 **No. 31,** 533: 7.

